# A de novo whole gene deletion of XIAP detected by exome sequencing analysis in very early onset inflammatory bowel disease: a case report

**DOI:** 10.1186/s12876-015-0394-z

**Published:** 2015-11-18

**Authors:** Judith R. Kelsen, Noor Dawany, Alejuandro Martinez, Christopher M. Grochowski, Kelly Maurer, Eric Rappaport, David A. Piccoli, Robert N. Baldassano, Petar Mamula, Kathleen E. Sullivan, Marcella Devoto

**Affiliations:** Division of Gastroenterology, Hepatology, and Nutrition, The Children’s Hospital of Philadelphia, Philadelphia, PA USA; Department of Biomedical Health Informatics, The Children’s Hospital of Philadelphia, Philadelphia, PA USA; Department of Pathology and Laboratory Medicine, The Children’s Hospital of Philadelphia, Philadelphia, PA USA; Division of Immunology and Allergy, The Children’s Hospital of Philadelphia, Philadelphia, PA USA; Nucleic Acid/PCR Core, The Children’s Hospital of Philadelphia, Philadelphia, PA USA; Division of Human Genetics, The Children’s Hospital of Philadelphia, Department of Pediatrics, Department of Biostatistics and Epidemiology, Perelman School of Medicine, University of Pennsylvania; Department of Molecular Medicine, University Sapienza, Rome, Italy; 7NW, Division of Pediatric Gastroenterology, 3400 Civic Center Blvd, The Children’s Hospital of Philadelphia, Philadelphia, PA 19104 USA

**Keywords:** VEO-IBD (very early-onset IBD), XIAP (x-linked inhibitor of apoptosis), XLP2 (X-linked lymphoproliferative Disease 2), WES (whole exome sequencing)

## Abstract

**Background:**

Children with very early-onset inflammatory bowel disease (VEO-IBD), those diagnosed at less than 5 years of age, are a unique population. A subset of these patients present with a distinct phenotype and more severe disease than older children and adults. Host genetics is thought to play a more prominent role in this young population, and monogenic defects in genes related to primary immunodeficiencies are responsible for the disease in a small subset of patients with VEO-IBD.

**Case Presentation:**

We report a child who presented at 3 weeks of life with very early-onset inflammatory bowel disease (VEO-IBD). He had a complicated disease course and remained unresponsive to medical and surgical therapy. The refractory nature of his disease, together with his young age of presentation, prompted utilization of whole exome sequencing (WES) to detect an underlying monogenic primary immunodeficiency and potentially target therapy to the identified defect. Copy number variation analysis (CNV) was performed using the eXome-Hidden Markov Model. Whole exome sequencing revealed 1,380 nonsense and missense variants in the patient. Plausible candidate variants were not detected following analysis of filtered variants, therefore, we performed CNV analysis of the WES data, which led us to identify a *de novo* whole gene deletion in XIAP.

**Conclusion:**

This is the first reported whole gene deletion in XIAP, the causal gene responsible for XLP2 (X-linked lymphoproliferative Disease 2). XLP2 is a syndrome resulting in VEO-IBD and can increase susceptibility to hemophagocytic lymphohistocytosis (HLH). This identification allowed the patient to be referred for bone marrow transplantation, potentially curative for his disease and critical to prevent the catastrophic sequela of HLH. This illustrates the unique etiology of VEO-IBD, and the subsequent effects on therapeutic options. This cohort requires careful and thorough evaluation for monogenic defects and primary immunodeficiencies.

## Background

Primary immunodeficiencies are a heterogeneous group of disorders that range in severity and clinical presentation, and may also lead to immune dysregulation such as severe inflammatory bowel disease (IBD), most frequently in patients with very early-onset IBD (VEO-IBD). VEO-IBD presents with a unique phenotype, including severe disease that is often unresponsive to conventional therapies. Disease causing variants have been detected in *IL-10*, *IL-10R* [1–4], *CYBB, CYBA, NCF1, NCF2 and NCF4* (chronic granulomatous disease), *FOXP3*, *WAS, MEFV, ITGB2*, as well as other genes in patients with severe VEO-IBD [3, 5–7]. In addition, mutations have been identified in X-linked inhibitor of apoptosis (XIAP) in patients with VEO-IBD [8]. While defects in the gene *SH2D1A* were initially detected in patients with X-linked lymphoproliferative disease, known as XLP1 [9, 10], mutations in *XIAP* result in XLP2 [11]. IBD can be the first and only feature of XLP2. We report here a case of a male who presented in the neonatal period with severe refractory IBD, without any evidence of immunodeficiency, and was found to have a *de novo* whole gene deletion of *XIAP* at age 17. This is the first reported whole gene deletion identified in the literature and illustrates the unique pathogenesis of disease in children with VEO-IBD.

## Case Presentation

**Fig. 1:**
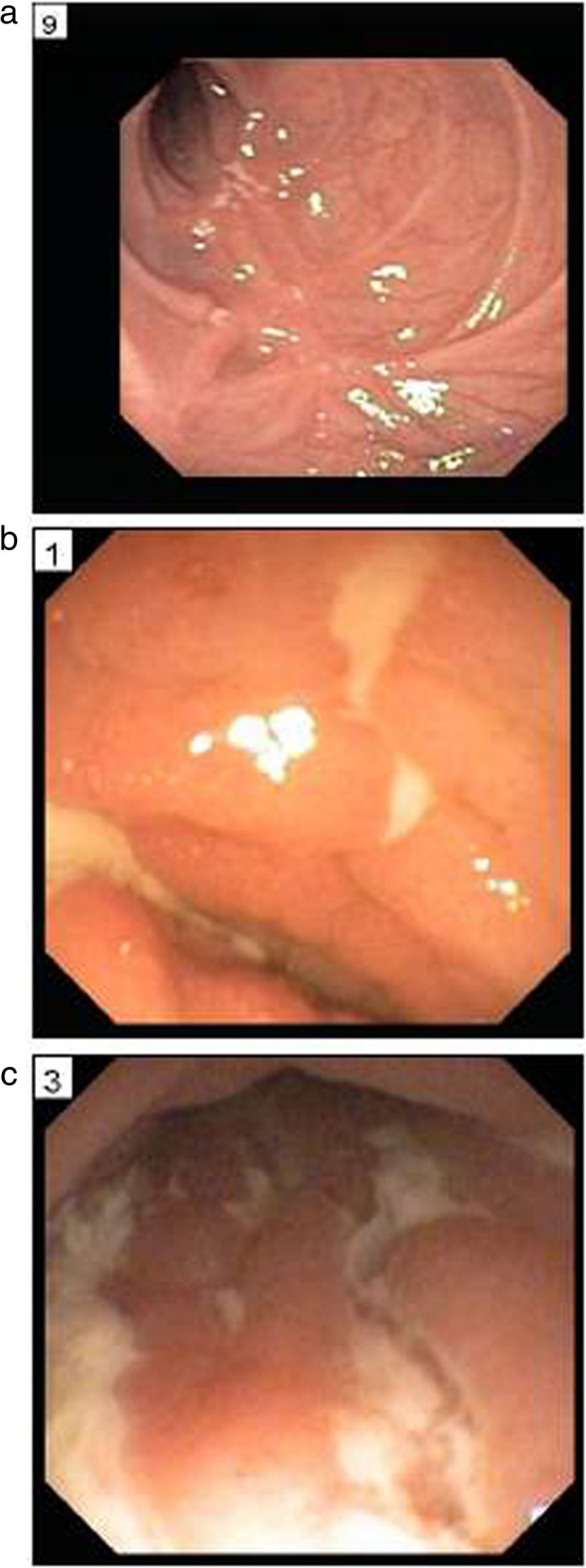
Endoscopy images: (**a**) duodenal narrowing; Ileoscopy: (**b**) and (**c**) Ulcerations and inflammation of the ileum

The patient presented with VEO-IBD at 3 weeks of life with severe, bloody diarrhea. His initial endoscopy and colonoscopy, performed at 8 weeks, demonstrated upper tract disease (gastric and duodenal) and severe pancolitis. At 5 months of age, he developed a perianal sinus tract. He was initially treated with Azulfidine, steroids and elemental formula. The patient was unable to tolerate oral feeds and was thus TPN (total parental nutrition) dependent. He continued to have profound growth failure (<5 %) and due to lack of response to the addition of antibiotics and enemas to his regimen, a G-tube was placed and Azathioprine was initiated. His course remained severe, complicated by pathological vertebral fracture. Repeat endoscopy at 2 years of age demonstrated severe upper tract disease and worsening colonic disease. His perianal disease progressed as well, with abscess and fistula formation. At approximately 4 years of age he was found to have a distal rectal stricture requiring repeated dilations. He was initiated on infliximab at 5 years of age in addition to azathioprine as dual therapy, which was changed to methotrexate a year later. At 7 years of age, during a rectal dilation he developed a colonic perforation and required a diverting ileostomy. The patient continued to be medically refractory, therefore, when he was 10 years old he underwent a colectomy. Post-operatively, his disease remained severe, and was unresponsive to medical therapy, including adalimumab, methotrexate, antibiotics, IVIG and finally vedolizumab, Repeat endoscopy performed demonstrated duodenal stricture and severe ileal disease (Fig. 1). In addition, the patient developed recurrent skin and intestinal abscesses and had significant steroid exposure throughout this time.

This patient’s young age at diagnosis and progressive disease severity prompted an immunologic evaluation to detect primary immunodeficiencies associated with VEO-IBD. At age 16, further studies including quantitative immunoglobulins, vaccine titers, B and T cell lymphocyte subset analysis, natural killer (NK) cells, and toll like receptors were obtained. He was found to have normal levels of circulating T cells, but mildly low subsets of NK cells. His B cells exhibited activation as detected by CD80 expression and he had increased plasmablasts (4 %) and low IgM memory B cells (3 %).

In addition, when he was 17 years old, after written informed consent was obtained from his parents and assent from the patient, the patient and his family were recruited in the VEO-IBD research study using whole exome sequencing (WES) to detect rare or novel variants that contribute to disease at our institution.

Exome sequencing of the patient and his mother were performed under protocol 2002-07-2805 that was approved by the Institutional Review Board at The Children’s Hospital of Philadelphia (CHOP). Exome capture was performed using the Agilent SureSelect V4 kit then sequenced using the Illumina HiSeq sequencer at an average coverage depth of 100X. Paired-end reads were aligned to the human reference genome GRCh37.p10 using Novoalign (V2.07.18; http://www.novocraft.com). Variants were detected using The Broad Institute’s GATK [12] best practices, then annotated using SNPEff [13], 1000 Genomes Project (www.1000genomes.org/), Exome Variant Server (EVS) (http://evs.gs.washington.edu/EVS/), the Exome Aggregation Consortium (ExAC) (http://exac.broadinstitute.org; release 0.3), and the Combined Annotation-Dependent Depletion tool (CADD) [14]. Identified variants were then filtered, retaining only nonsynonymous missense and nonsense, rare (minor allele frequency <0.1 % in all 3 databases) or novel mutations, with a CADD score ≥10. Variants were further restricted to genes involved in primary immunodeficiency. Copy number variation (CNV) analysis was performed using the eXome-Hidden Markov Model (XHMM) [15].

Genotyping was conducted using the Illumina HumanHap550v3.0 Beadchip. The data was analyzed using Beadstudio software (Illumina) to confirm the deletion that was found in the WES data.

The *XIAP* CNV was confirmed using the Bio-Rad QX100 droplet digital PCR (ddPCR) platform. Predesigned primer/probe configurations were used for *XIAP* (cat#: Hs04517714_cn) and the reference gene *TERT* (cat#4403326). Standard ddPCR conditions and analysis methods were used for this assay [16].

Whole exome sequencing revealed 1,380 nonsense and missense variants in the patient. However, after restricting the variants to rare or novel variants in genes associated with immunodeficiency, only a single variant was identified in CCHCR1 (p.Gln609Pro) that met our criteria. The same variant was also found in the patient’s unaffected mother and therefore was unlikely to be associated with his disease.

**Fig. 2:**
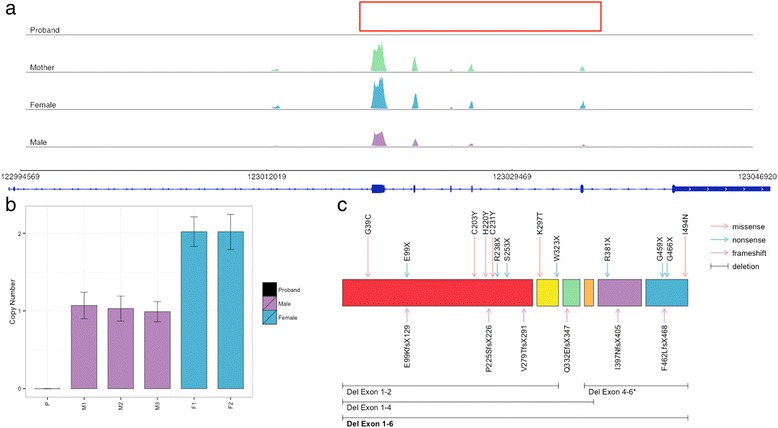
XIAP whole gene deletion. **a** Depth of coverage of XIAP exons from whole exome sequencing data in the proband, his mother (green), a female control (blue) and a male control (purple). The red box indicates missing coverage for XIAP exons in the proband. **b** Copy number analysis of XIAP using ddPCR shows gene deletion in the proband compared to control males (purple) and females (blue). **c** Location of the XIAP whole gene deletion (bold) and previously reported XIAP mutations associated with IBD across the 6 coding exons of XIAP. *Exon numbers adjusted to reflect coding exons of XIAP

We then performed CNV analysis of the WES data of the patient and his mother, which led us to identify a *de novo* whole gene deletion in *XIAP* (Fig. 2a). The deletion was then validated using both SNP arrays and ddPCR (Fig. 2b). The SNP array deletion spanned from rs5958318 at position chrX:122,992,832, near the 5’ end to rs10521711 at position chrX:123,047,926 near the 3’ UTR of *XIAP*, which confirmed that the entire gene was deleted.

Functional analysis of *XIAP* demonstrated lack of *XIAP* expression in CD4+ and CD8+ T cells, NK cells and B cells. Following identification of this deletion the patient was referred to our Bone Marrow Transplant Team for a bone marrow transplantation (BMT).

## Discussion

Monogenic defects of primary immunodeficiency can present as severe neonatal IBD, including epithelial barrier defects, phagocyte defects (Chronic granulomatous disease, defects of NADPH oxidase LAD), B and T cell abnormalities (Severe combined immunodeficiency, common variable immunodeficiency, Wiskott-Aldrich Syndrome, immunodysregulation polyendocrinopathy enteropathy X-linked syndrome), autoimmunity (including XLP2) and IL-10R defects. Due to this patient’s severe refractory disease and age of onset, evaluation for primary immunodeficiency was performed. Although the immune work up was unrevealing, evaluation of WES data demonstrated a whole gene deletion of XIAP.

X-linked lymphoproliferative disease, XLP1, first described in 1974 by Purtilio, results in the immunodeficiency of severe susceptibility to EBV due to defects in NK cell mediated toxicity, and leads to hemophagocytic lymphohistocytosis (HLH), dysgammaglobulimemia and lymphoma [17]. This triad is secondary to inactivating mutations in *SH2D1A*, encoding SLAM-associated protein (SAP), on chromosome Xq25 [10]. In 2006, Rigaud et al. demonstrated that mutations in the gene that encodes the X-linked inhibitor-of-apoptosis XIAP (also termed BIRC4) resulted in another X-linked lymphoproliferative syndrome, XLP2, distinct from SAP deficiency XLP (XLP1) [11]. The immune dysregulation in these patients, including our patient described here, was characterized by absent XIAP in lymphocytes, NK cells and myeloid cells. Two of the patients described by Riguard had hemizygous mutations in *XIAP* and developed IBD. Worthey et al. first identified XIAP deficiency in VEO-IBD, in a 15-month-old male with severe fistulizing Crohn’s disease. This patient had a novel hemizygous mutation and ultimately underwent BMT, which proved to be curative [8]. Since then, several other cases of *XIAP* missense or nonsense mutations in patients with VEO-IBD have been reported [18]. Zeissig et al. further demonstrated the role of XIAP deficiency in severe VEO-IBD, with the identification of variants in 4 male pediatric patients, ages 1–16 [18]. The variant identified in the patient described in this study is the first whole gene deletion, to our knowledge, to be reported in VEO-IBD, and its identification provided a potentially curative therapeutic option. The mapping of the deletion described in this study compared to mutations previously reported in the literature in association with IBD is depicted in Fig. 2c.

The inhibitors of apoptosis (IAPs) genes, such as *XIAP*, are instrumental for both innate and adaptive immunity. XIAP inhibits apoptosis in activated T cells, thus allowing expansion and survival via blockade of initiator and effector caspases [19] or through binding to death-inducing caspases and subsequent proteosomal mediated degradation. IAPs contain 1–3 baculoviral IAP repeat (BIR) homology domains, a ubiquitin binding domain (UBA) and a C-terminal RING domain [20]. BIR2 and BIR3 domains of cIAP1, CIAP2 and *XIAP* are required for binding to and suppression of specific cell death-inducing caspases [19]. XIAP inhibits apoptosis by binding to and blocking activated forms of caspases 3,7 and 9. *XIAP* participates in innate immunity through signaling pathways and cellular responses via ubiquitin ligases activity [20]. It is required in the signaling and function of the pattern recognition receptors, *NOD1*, *NOD2* and Dectin-1 [19]. Upon activation during infection, *NOD1/2* and Dectin-1 promote cytokine production, with subsequent clearance of pathogens. *XIAP* is also involved in the inhibitory signaling of inflammasomes (*NLRP3* in murine models) and decreases TNF-α activation by pathogen-associated molecular patterns (PAMPs). Therefore, *XIAP* eradicates local acute inflammation and maintains low levels of baseline inflammation. In XIAP-deficient patients, both arms of the immune system, innate and adaptive responses, are affected, with a cyclical environment of unchecked activation of inflammasomes and intestinal permeability to pathogens leading to the accumulation of pro-inflammatory cytokines such as TNF-α, IL-1β and IL-18. Finally, there is increased cell-death of cells, including lymphocytes and myeloid cells and chronic inflammation. Ultimately the patient develops the phenotype of splenomegaly, IBD and HLH [20].

## Conclusion

Due to the extensive differential in the work up of our patient with severe VEO-IBD, next generation sequencing technology can be of benefit. Targeted sequencing, to evaluate for identified monogenic defects in VEO-IBD, or WES to study the whole exome, can offer a quicker method of analysis rather than a gene-by-gene approach. The results of this critical finding directed the course of action of treatment. Because XIAP deficiency has a high risk of fatal HLH, and due to his severe disease, he has undergone bone marrow transplantation. While XLP2 functional analysis currently exists, the wide differential in this case made WES, and the multidisciplinary approach involving gastroenterology, genetics, immunology and bioinformatics an attractive and effective option.

## Consent

Written informed consent was obtained from the patient’s parents as he is a minor, for publication of this Case report and any accompanying images. A copy of the written consent is available for review by the Editor of this journal.

## Abbreviations

BMT: bone marrow transplantation
CADD: combined annotation-dependent depletion
CHOP: The Children’s Hospital of Philadelphia
CNV: Copy number variation analysis
DHR: dihydrorhodamine
EVS: Exome Variant Server
ExAC: Exome Aggregation Consortium
HLH: hemophagocytic lymphohistocytosis
IAP: inhibitors of apoptosis
IBD: inflammatory bowel disease
MAF: minor allele frequency
NK: natural killer cells
PAMP: pathogen-associated molecular patterns
TPN: total parental nutrition
Sap: SLAM-associated protein
VEO-IBD: very early-onset inflammatory bowel disease
WES: whole exome sequencing
XHMM: eXome-Hidden Markov Model
XIAP: X-linked inhibitor-of-apoptosis
XLP1: X-linked lymphoproliferative disease
XLP2: X-linked lymphoproliferative Disease 2
