# Erratum to: A de novo whole gene deletion of XIAP detected by exome sequencing analysis in very early onset inflammatory bowel disease: a case report

**DOI:** 10.1186/s12876-015-0412-1

**Published:** 2015-12-18

**Authors:** Judith R. Kelsen, Noor Dawany, Alejandro Martinez, Christopher M. Grochowski, Kelly Maurer, Eric Rappaport, David A. Piccoli, Robert N. Baldassano, Petar Mamula, Kathleen E. Sullivan, Marcella Devoto

**Affiliations:** 1 Division of Gastroenterology, Hepatology, and Nutrition, The Children’s Hospital of Philadelphia, Philadelphia, PA USA; 2 Department of Biomedical Health Informatics, The Children’s Hospital of Philadelphia, Philadelphia, PA USA; 3 Department of Pathology and Laboratory Medicine, The Children’s Hospital of Philadelphia, Philadelphia, PA USA; 4 Division of Immunology and Allergy, The Children’s Hospital of Philadelphia, Philadelphia, PA USA; 5 Nucleic Acid/PCR Core, The Children’s Hospital of Philadelphia, Philadelphia, PA USA; 6 Division of Human Genetics, The Children’s Hospital of Philadelphia, Department of Pediatrics, Department of Biostatistics and Epidemiology, Perelman School of Medicine, University of Pennsylvania; Department of Molecular Medicine, University Sapienza, Rome, Italy; 7 7NW, Division of Pediatric Gastroenterology, The Children’s Hospital of Philadelphia, 3400 Civic Center Blvd, Philadelphia, 19104 PA USA; 8 Department of Pediatrics, University of Pennsylvania, Philadelphia, USA; 9 Department of Molecular Medicine, University Sapienza, Rome, Italy; 10 Department of Biostatistics and Epidemiology, Perelman School of Medicine, University of Pennsylvania, Philadelphia, USA

Following publication of the original version [1] of the article in *BMC Gastroenterology* it was brought to our attention that the author’s name has been incorrectly spelt, instead of ‘Alejuandro’ it should be spelt ‘Alejandro’.
